# Systematic Identification and Validation of Suitable Reference Genes for the Normalization of Gene Expression in *Prunella vulgaris* under Different Organs and Spike Development Stages

**DOI:** 10.3390/genes13111947

**Published:** 2022-10-25

**Authors:** Hui Zheng, Hongguang Zhao, Xuemin Zhang, Zongsuo Liang, Qiuling He

**Affiliations:** 1 Key Laboratory of Plant Secondary Metabolism and Regulation of Zhejiang Province, College of Life Science and Medicine, Zhejiang Sci-Tech University, Hangzhou 310018, China; 2 Tasly Botanical Pharmaceutical Co., Ltd., Shangluo 726000, China; 3 Tasly R&D Institute, Tasly Holding Group Co., Ltd., Tianjin 300410, China; 4 Shaoxing Academy of Biomedicine, Zhejiang Sci-Tech University, Shaoxing 312000, China

**Keywords:** *Prunella vulgaris* L., reference gene, data normalization, RT-qPCR, Spica Prunellae, medicinal herb

## Abstract

The quantitative real-time PCR (qRT-PCR) is an efficient and sensitive method for determining gene expression levels, but the accuracy of the results substantially depends on the stability of the reference gene (RG). Therefore, choosing an appropriate reference gene is a critical step in normalizing qRT-PCR data. *Prunella vulgaris* L. is a traditional Chinese medicine herb widely used in China. Its main medicinal part is the fruiting spike which is termed Spica Prunellae. However, thus far, few studies have been conducted on the mechanism of Spica Prunellae development. Meanwhile, no reliable RGs have been reported in *P. vulgaris.* The expression levels of 14 candidate RGs were analyzed in this study in various organs and at different stages of Spica Prunellae development. Four statistical algorithms (Delta Ct, BestKeeper, NormFinder, and geNorm) were utilized to identify the RGs’ stability, and an integrated stability rating was generated via the RefFinder website online. The final ranking results revealed that *eIF-2* was the most stable RG, whereas *VAB2* was the least suitable as an RG. Furthermore, *eIF-2* + *Histon3.3* was identified as the best RG combination in different periods and the total samples. Finally, the expressions of the *PvTAT* and *Pv4CL2* genes related to the regulation of rosmarinic acid synthesis in different organs were used to verify the stable and unstable RGs. The stable RGs in *P. vulgaris* were originally identified and verified in this work. This achievement provides strong support for obtaining a reliable qPCR analysis and lays the foundation for in-depth research on the developmental mechanism of Spica Prunellae.

## 1. Introduction

Gene expression refers to the amount of mRNA expression of a particular gene in a tissue or cell at a specific time. At present, the analysis methods of plant gene expression include Southern hybridization, Northern hybridization, in situ hybridization, traditional PCR, and qRT-PCR [1]. Among them, the qRT-PCR completes an expression analysis by detecting real-time fluorescent signal changes during the entire PCR reaction, and it has received a lot of attention in molecular biology research because of its excellent accuracy, sensitivity, specificity, and cost-effectiveness [2,3]. However, a number of variables, such as RNA integrity, purity, quality, reverse transcription efficiency, primer performance, normalization, and a host of other elements, might affect how accurate the data on expression are [4,5]. In order to control variables and ensure the accuracy of results, selecting one or more stable RGs is crucial, especially for samples with large fluctuations of expression levels [6]. Using inappropriate RGs to normalize the expression of additional genes in an RT-qPCR analysis may lead to misunderstandings about the expression level of the target gene [2,7,8]. Housekeeping genes are essential to maintain the fundamental biological processes of cells and can be expressed stably in cells, so they are often used as internal RGs [1,9]. The expression levels of ideal RGs in plants will not change significantly with the changes in external conditions, samples, and treatment methods [10]. However, research has demonstrated that the expression of RGs is not always steady under certain experimental conditions [11]. That is, given specific experimental circumstances, the stable expression of any housekeeping gene is only consistent in a restricted range of cells [11]. Thus, choosing the appropriate RGs is crucial when using a qPCR to evaluate the gene expression level of plants under particular circumstances.

The expression of the internal RG is relatively constant across tissues and cells, so it is often used as a reference when detecting the change in gene expression levels [12]. Its role is to correct for the experimental errors that exist in the amount and process of sample loading, so as to ensure the accuracy of the experimental results [13]. Theoretically, RGs are genes that are constantly expressed in all types of tissues and cells under all experimental conditions. However, studies have demonstrated that the expression level of conventionally employed RGs is variable based on different experimental conditions and materials [11]. As a result, RGs tend to vary amongst different materials [6]. Housekeeping genes are essential for the maintenance of basic cellular activities and are often involved in processes such as cytoskeleton building, vesicle transport, glycolysis, protein synthesis, and protein degradation [14]. Traditionally, glyceraldehyde-3-phosphate dehydrogenase (*GAPDH*), β-actin (*ACT*), 18S ribosomal RNA (*18S rRNA*), 28S ribosomal RNA (*28S rRNA*), ubiquitin (*UBQ*), cyclophilin (*CYP*), tubulin β (*TUB*), elongation factor 1-α (*EF1A*), protein phosphatase 2A regulatory subunit (*PP2A*), and tubulin α (*TUA*) are genes that play a housekeeping role in cells and are often used as RGs for the standardization of qPCR data [15,16]. Previous studies on screening endogenous genes have focused on model plants, major food crops, and some cash crops. Nevertheless, as the therapeutic potential of medicinal plants is becoming more widely recognized, it is essential to investigate their molecular mechanisms and identify the suitable RGs under various experimental settings. In this process, many new genes have also been determined as appropriate RGs. Currently, some medicinal plants have performed the study of internal RG selection and validation, including *Angelica sinensis* [17], *Desmodium styracifolium Merr* [18], *Rubus* [19], *Schima superba* [1], and *Isatis indigotica fortune* [20].

An expression stability analysis of each candidate gene is the focus of internal RG screening. Currently, there are five major statistical algorithms for identifying the optimal RGs which can be stably expressed in specific conditions: geNorm [21], NormFinder [22], BestKeeper [23], ΔCt method [24], and RefFinder [25]. The geNorm algorithm computes the M value of the stability of each RG to select the most stable endogenous gene, and the smaller the M value, the more stable the RG is, and vice versa. geNorm also calculates the pairwise variation V value of the RGs and determines the optimal number of endogenous genes depending on the value of V_n_/V_n+1_. The optimal number of endogenous genes is n when V_n_/V_n+1_ is <0.15, which is the default value for V. There should be n + 1 endogenous genes if V_n_/V_n+1_ > 0.15. The NormFinder algorithm will filter the most appropriate RGs according to the stability value, and the most appropriate RG will be the one with the smallest stability value. The NormFinder program can evaluate the most stable endogenous genes not only within groups but also between groups. The BestKeeper software was used to calculate each gene’s correlation coefficient (r), standard deviation (SD), and coefficient of variation (CV). The internal RG is more stable when r is higher, while SD and CV are lower, and vice versa. Furthermore, the internal RG exhibits unstable expression when SD > 1. This procedure can evaluate the stability of the RGs and the degrees of expressiveness of different target genes. The ΔCt method is performed to make a two-by-two comparison by calculating the SD of each pair of candidate internal RGs and the mean SD value of each gene to finally identify the more stable internal RGs. RefFinder is a web tool for the comprehensive ranking of candidate gene stability based on the geNorm, NormFinder, BestKeeper, and Delta Ct algorithms. Each evaluation software has its merits, and the results will be more accurate when used together. Their help identified the optimal RGs for many species, such as *D. styracifolium* [18], *Punica granatum* L. [26], and *Oryza sativa* L. [27].

Flower bud differentiation is the process by which the growing point of a plant stem changes from meristematic leaves and axillary buds to inflorescences or flowers. Flower bud differentiation is an important stage that determines the development of Spica Prunellae, and it is a complex process that arises in response to the integration of signals from the external environment and internal factors [28]. The Spica Prunellae develops once a year, usually starting from the end of April and ending in the beginning of June, with a total differentiation period of nearly 40 days.

**Figure 1:**
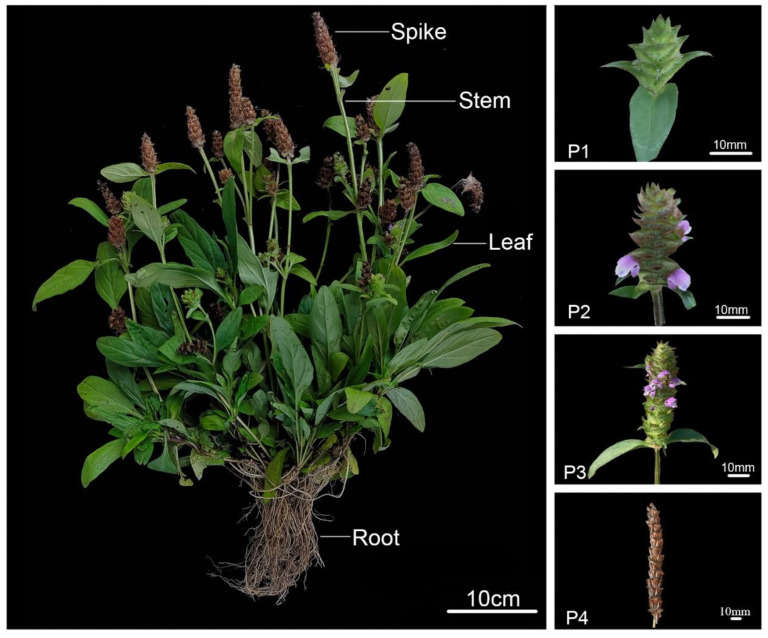
Intact plants and four distinct developmental stages of Spica Prunellae. P1: bolting stage; P2: early flowering stage; P3: complete flowering stage; P4: mature stage.

*P. vulgaris* is a low-growing perennial herbaceous plant widely distributed across northeastern Asia, such as in China, Japan, and Korea. Among them, Henan, Anhui, Jiangsu, Hunan, and other provinces are the main producing areas in China [29]. *P. vulgaris* has a wide range of medicinal values, and the whole plant can be used as medicine. The principal medicinal part of the plant is the dried mature spike, which has been in use for thousands of years in China. Different organs (root, stem, leaf, and spike) of *P. vulgaris* are sold at different prices in the market, among which the spike has the highest medicinal value and economic benefits. Recent reports on *P. vulgaris* have mostly concentrated on its pharmacological properties and clinical applications. Modern pharmacological and biological studies showed that *P. vulgaris.* exhibits numerous functions, including antibacterial, anticancer [30], anti-inflammatory [31], antiviral [32], immune-enhancing [33], antioxidant [34], antiproliferative [35], antihyperlipidemic [36], and free radical scavenging activities [37]. However, the molecular mechanism of its flower spike development is still unclear. With the development of molecular biology, the research to reveal the molecular mechanism of Spica Prunellae development has become increasingly intense. At the same time, different materials typically contain various stable genes that may be expressed at different expression levels at different periods of growth and development. Therefore, screening appropriate internal RGs in different development periods and organs in *P. vulgaris* is highly important. However, so far, there are no reports on this aspect of endogenous gene screening in *P. vulgaris*. In total, 14 commonly used candidate RGs: Actin (*ACT7*, *ACT12*, *ACT1*), *18S rRNA*, Translation initiation factor (*eIF-3*, *eIF4A-III*, *eIF-2*), Histone (*His3.3*), α-tubulin (*TUA6*), Cyclophilin (*CYP38*), Protein phosphatase 2Asubunit (*PP2A-2*, *PP2A-3*, *PP2A- 4*), and Homeodomain transcription factor (*VAB2*) were selected for this study based on the analysis of our previous transcriptome datasets in *P. vulgaris.* The expression levels of candidate RGs in different organs (root, stem, leaf, and spike) of *P. vulgaris* and the Spica Prunellae at different developmental stages (heading stage, early flowering stage, full-flowering stage, and ripening stage) were detected by the qPCR technique (Figure 1). Four alternative statistical algorithms—Delta CT, geNorm, NormFinder, and BestKeeper—were employed to evaluate the stability of the fourteen genes’ expression levels. The online software RefFinder was also used to carry out a thorough ranking of these RGs’ stability under each unique experimental scenario. The expression of *PvTAT* and *Pv4CL2*, genes regulating the synthesis of rosmarinic acid in *P. vulgaris*, were studied throughout several organs to further verify the reliability of the screened RGs. These findings will hopefully provide an important reference for further investigation into the molecular mechanism of Spica Prunellae development.

## 2. Materials and Methods

### 2.1. Plant Materials

*P. vulgaris* seeds obtained from Shangluo Tasly Pharmaceutical Co., Ltd., Shaanxi Province, were selected for this study. Plants were cultivated in the artificial climate chamber of Zhejiang Sci-Tech University (120°35′ E; 30°31′ N), Zhejiang, China. The cultivation conditions were programmed for a 16 h day (25 °C) and 8 h night (20 °C) cycle with 60% relative humidity. According to the established phenological period of *P. vulgaris*, Spica Prunellae at different growth stages (bolting stage, early flowering stage, complete flowering stage, and mature stage) and different organs (root, stem, leaf, and spike) of *P. vulgaris* in the complete flowering stage were collected [38]. The gathered samples were washed with distilled water, dried with absorbent papers quickly, liquid nitrogen-snap frozen, then kept at −80 °C for subsequent use. Three biological replicates were adopted in the stability analysis for each sample and time point, and five biological replicates were applied to the validation.

### 2.2. Total RNA Extraction and cDNA Synthesis

Total RNAs were extracted from samples using the RNAprep Pure Plant Plus Kit DP441 (Tiangen Biotech, Beijing, China) according to the manufacturer’s protocol and then treated with DNase I (Tiangen Biotech) to eliminate contaminating DNA. The concentration and purity of RNA were measured using a NanoDrop™ 2000 spectrophotometer (Thermo, Waltham, MA, USA), and the integrity of the RNA was confirmed by 1.5% (*w*/*v*) agarose gel electrophoresis. For the purpose of the cDNA synthesis, only RNA samples with content greater than 150 ng/µL, an A260/A280 ratio ranging from 1.8 to 2.2, and an A260/A230 ratio greater than 1.8 were required. The reverse transcription kits TaKaRa RR036A (TaKaRa Bio Inc., Dalian, China) were utilized to create the first-strand cDNA from 1 μg of RNA for 20 µL of reaction with oligo dT primers. To perform the qPCR, the acquired cDNA was subsequently diluted using RNase Free ddH_2_O.

### 2.3. Selection and Validation of Candidate RGs and the Design of qPCR Primers

In total, 25 candidate RGs were selected after a review of the research literature on plant RGs. The transcriptome sequences of *P. vulgaris* were retrieved from GenBank (accession number: SRR7873856). To ensure the accuracy and reliability of the screened RGs, the CDS sequence of Arabidopsis was obtained from GenBank and used as the query sequence in BlASTn to locate the homologous sequence from *P. vulgaris*. Finally, 14 candidate RG sequences (*ACT7*, *ACT12*, *ACT1*, *18S rRNA*, *eIF-3*, *eIF4A-III*, *eIF-2*, *His3.3*, *TUA6*, *CYP38*, *PP2A-2*, *PP2A-3*, *PP2A-4*, and *VAB2*) with the highest homology, the e-values all less than 1 × 10^−5^, and the alignment rates ranged from 78.01% to 95.58% were acquired. The quantitative primers of the candidate RGs were designed by Primer-BLAST (https://www.ncbi.nlm.nih.gov/tools/primer-blast/, accessed on 27 November 2021) with a manual inspection. The parameters were as follows: primer length 18–25 bp, amplification product size 150–200 bp, GC content 45–65%, melting temperature 58–60 °C, no hairpin structure, homodimer, and heterodimer (Table 1). The designed primers were synthesized by Youkang Biotechnology Co., Ltd. (Hangzhou, Zhejiang, China). A melting curve analysis was used to evaluate the specificity of each primer pair (Figure S2), and the size of each amplicon was determined by electrophoresis on a 1.5% agarose gel (Figure S1). A ten-fold dilution of cDNA was used to assess the primer efficiency, and the amplification efficiency (E) and correlation coefficient (R^2^) of the primers were then computed using the established standard curve (Figure S3). The slope of the curve was used to determine the E of the primers based on the theoretical formula E = [10^(−1/slope)^ − 1] × 100% [39,40].

### 2.4. Quantitative Real-Time PCR (qRT-PCR) Analyses

The qPCR reactions were carried out using TB Green Premix Ex Taq II (TaKaRa Bio Inc., Dalian, China) in 96-well plates with an ABI 7500 Real-Time PCR System (Applied Biosystems, Waltham, MA, USA). Each PCR reaction mixture (final volume of 20 µL) consisted of TB Green Premix Ex Taq II (5 µL), each forward, and reverse primers with a concentration of 10 μM (0.4 µL), RNase-free dH_2_O (2.6 µL), ROX II (0.1 µL), and 1.5 µL of the cDNA template (diluted ten times with RNase-free ddH_2_O). All of the RGs in every run were kept in NTC (no template control). All experiments were maintained in three technical replicates and three to five biological replicates. The PCR amplification cycles’ conditions: 95 °C for 10 min, followed by 40 cycles of 95 °C for 5 s, and 58 °C for 30 s. A melt curve analysis was performed at 95 °C for 15 s, 60 °C for 1 min, and 95 °C for 15 s at the end of the PCR run. Ct values were recorded and taken for further analyses.

### 2.5. Analysis of Expression Stability of Candidate RGs

Utilizing Microsoft Excel 2019, a descriptive statistical analysis was carried out to assess the expression stability of potential RGs. Four different software programs (Delta Ct, NormFinder, geNorm, and BestKeeper) based on the experimental design and manufacturers’ instructions were used to ascertain the expression stability of 14 candidate RGs in various organs and experimental periods.

The raw Ct values obtained by qRT-PCR were transformed into relative expression levels for each RG using the formula 2^−ΔCT^ [41] (ΔCT = each corresponding Ct value—the lowest Ct value for that gene in different samples), which was subsequently employed for an additional analysis in geNorm and NormFinder. These values were imported into geNorm to calculate the variable M that gauges the consistency of gene expression. The M values were inversely correlated with the stability of the genes, meaning that the expression of this RG in the identified genes was the most stable when the M value was the lowest [21]. geNorm can also determine the paired variation V value of the normalization factor using the geometric mean from expression levels of the most stable RGs, and the value of V_n_/V_n+1_ can be used to calculate the optimal number of internal RGs (with a cut-off value of V_n+1_  <  0.15) [42].

NormFinder is a mathematical model describing the expressed stable values of RGs by computing the variance, both within-group variance and between-group [22]. Moreover, the stability value (SV) determines the ideal gene or set of genes for standardization.

The analysis of the Delta Ct and BestKeeper software employed raw Ct data. The Delta Ct has the ability to determine the standard deviations (SD) value of the Ct value of each selected RG. Lower RG stability is associated with greater SD values [41]. The BestKeeper software algorithm utilizes the coefficient of variance (CV), and the SD of the Ct value. The value of the correlation coefficient between the candidate gene determines the stability of the RGs [23]. Genes with higher stability typically have lower SD (lower than 1) and CV values.

In addition to these four algorithms, RefFinder (an online tool) was also used to generate an overall comprehensive ranking of RGs. The geometric mean was calculated for the final overall ranking of the genes by the RefFinder algorithm, which combined ranks from the Delta Ct, geNorm, NormFinder, and BestKeeper.

### 2.6. RGs Validation

To further confirm the dependability of the selected RGs, the expression patterns of *PvTAT* and *Pv4CL2*, genes involved in the controlling of the synthesis of rosmarinic acid in *P. vulgaris,* were analyzed throughout different organs. The relative expression levels of the target genes *PvTAT* and *Pv4CL2* were computed according to the formula 2^−ΔΔCt^ [43].

### 2.7. Data Analysis

Delta Ct, geNorm, NormFinder, and BestKeeper were used for the data analysis. Figures were created utilizing GraphPad Prism 6 (GraphPad Software, Inc., La Jolla, CA, USA). Microsoft Excel 2019 and SPSS 21.0 software were used for the statistical analysis.

## 3. Results

### 3.1. Selection of RGs and Analysis of Primer Amplification Specificity and Efficiency

Based on the previous studies, 14 genes were identified as potential RGs by mining the transcriptome data of *P. vulgaris* (GenBank accession: SRR7873856). The details of gene symbols, gene IDs, gene names, Arabidopsis homologous gene numbers, primer sequences, annealing temperature (°C), amplification length (bp), PCR efficiency (E), and correlation coefficient (R^2^) for the 14 candidate genes were shown in Table 1. Specific qPCR primers were created on the basis of the sequences of transcriptome data, and the specificity of the primers was evaluated by both the gel electrophoresis and melting curves. The results showed that all the internal RG primers were single bands in an agarose gel electrophoresis analysis, and the amplification products were consistent with the expected fragment size and without any dimers or non-specific amplification appearing (Figure S1). The identical outcomes for all of the primer sets with single amplification peaks were confirmed by a qRT-PCR melting curve analysis (Figure S2). The amplification efficiency of 14 pairs of primers varied between 98.37% and 108.49%, and all the correlation coefficients (R^2^) values were above 0.99 (Figure S3). Briefly, the outcomes suggested that the primers of these 14 genes were thoughtfully created with reasonable specificity and showed excellent amplification ability, suggesting that these primers were suitable for subsequent experiments.

### 3.2. Expression Profiles of Candidate RGs

**Figure 2:**
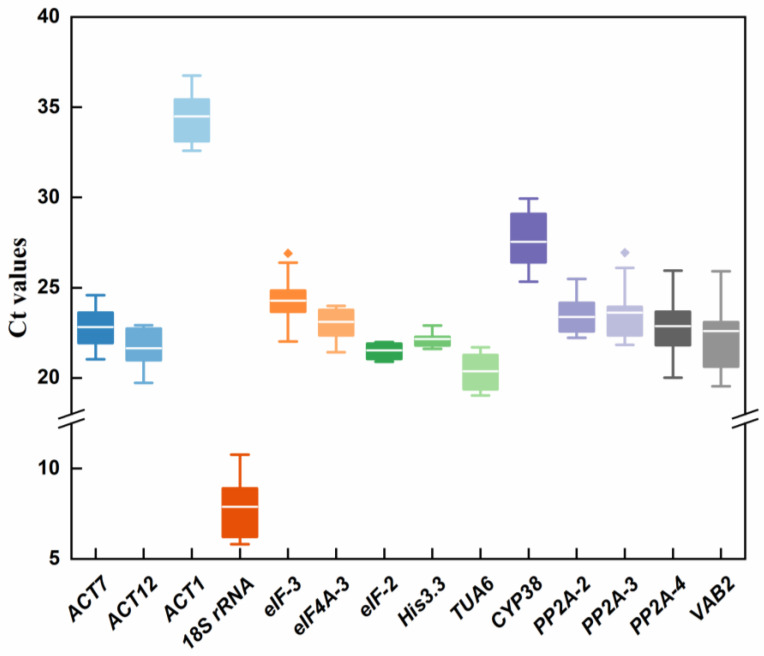
Expression levels of 14 candidate reference genes in all *P. vulgaris* samples. The box chart indicates the interquartile range. The outer box shows the 25th to 75th percentile, and the inner box indicates the average. Lines across the boxes depict the medians and whiskers represent the maximum and minimum values. The dots represent the outlier.

To evaluate the expression stability of 14 candidate internal RGs under different conditions, the mean cycle threshold values (Cps) were used to determine their transcript abundances, ranging from 5.81 to 36.74. The Cp value was inversely proportional to the transcription level of the gene. In other words, the expression abundance increased as the Cp value decreased. According to Figure 2, the mean Cp values of 14 RGs ranged from 7 to 35, with the bulk falling between 21 and 24. Across all samples, the lowest and highest Ct values appeared in *18S rRNA* and *ACT1* with 5.81–10.77 and 32.58–36.74, indicating that they were the most highly and lowly expressed genes, respectively. Among the 14 genes, *His3.3* (21.62–22.90) was expressed in high abundance with the small range of variation, while the expression levels of *VAB2* (19.53–25.90) were the most variable (Supplementary Table S1). Overall, the expression profiles of the endogenous genes were displayed through Ct values combined with the box plot, allowing us to have a glimpse of the gene stability. Our initial findings demonstrated that the expression levels of *His3.3*, *eIF-2*, *eIF4A-3*, *PP2A-2*, *ACT7*, and *eIF-3* were relatively stable and showed high expression levels, while the expression of *18S rRNA* and *VAB2* varied greatly.

### 3.3. Expression Stability Estimation of Candidate RGs by Five Bioinformatic Programs

For further proof that the RGs were stable, Spica Prunellae at different developmental stages (bolting stage, early flowering stage, full flowering stage, maturity stage) and various organs (leaf, stem, root, spike) at the full flowering stage were collected as samples.

All the samples were tested to assess the stability of the potential RGs by four algorithms: BestKeeper, geNorm, NormFinder, and Delta Ct. Finally, the RefFinder tool (https://blooge.cn/RefFinder/, accessed on 3 March 2022) was utilized to comprehensively rank the expression stability of all potential RGs to screen out the most suitable ones for subsequent research.

#### 3.3.1. geNorm Analysis

**Figure 3:**
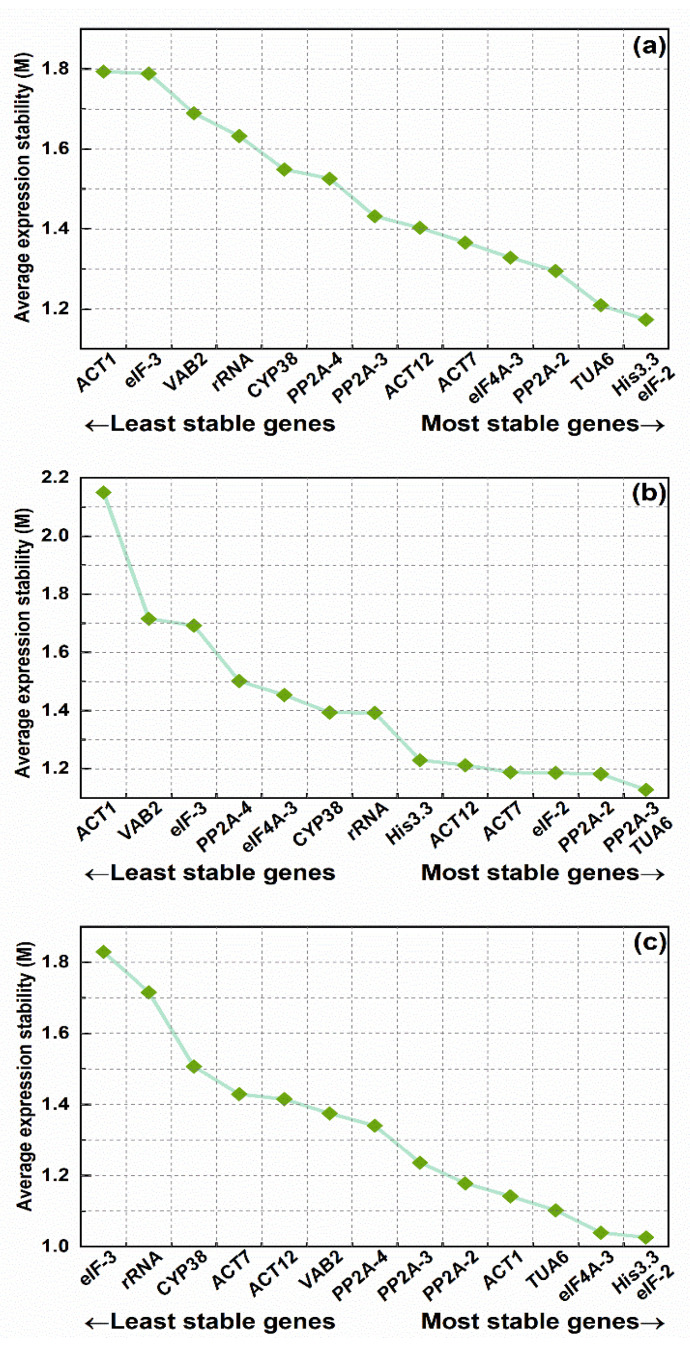
Gene expression stability and ranking of fourteen reference genes based on geNorm. The expression stability value (M) for each gene was obtained and graphed. The lower the M value, the more stable the expression. The most stable genes are on the right and the least stable genes are on the left. (**a**) Total samples: all root, stem, leaf, and spike samples of *P. vulgaris*; (**b**) different organs: root, stem, leaf, and spike samples during the full flowering stage; (**c**) different periods: Spica Prunellae from four stages: bolting stage, early flowering stage, full flowering stage, maturity stage.

The average expression stability measurement (M) value and paired variation (V) value calculated by the geNorm software algorithm were used to rank the gene expression stability and determine the optimal number of RGs for accurate normalization. The M value was negatively correlated with gene stability. The gene expression stability of the RGs increased with decreasing M value. Meanwhile, if the M value of a gene was greater than 1.5, it indicated that it was not suitable as an RG [21,44]. The results revealed that all the candidate internal RGs had different stability levels under different conditions (Figure 3). The M value of *eIF-2* was the lowest, whereas the M value of *ACT1* was the highest, indicating that *eIF-2* was the gene with the highest level of stability across all samples, while the *ACT1* was the least stable one. The optimal internal RG in the different periods was also *eIF-2*, while the most erratic gene was *eIF-3.* Notably, the steadiest RG was *TUA6* in an organ subset, while *ACT1* seemed to be less stable than other candidate RGs.

**Figure 4:**
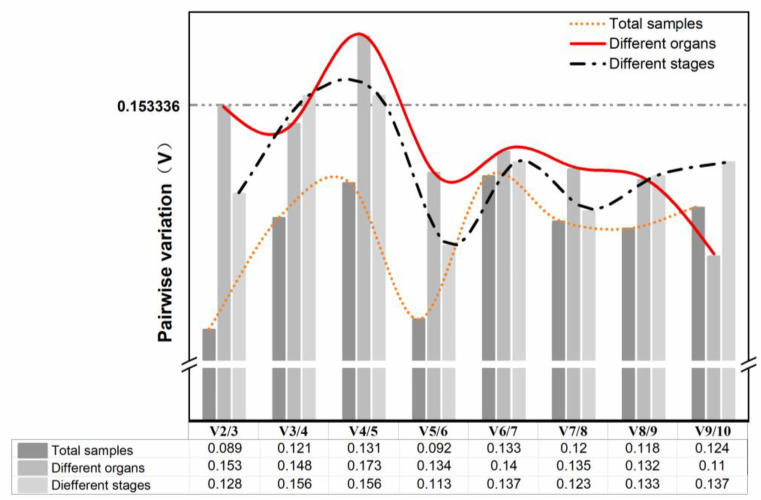
Pairwise variation (V) of the 14 candidate reference genes calculated by geNorm to determine the optimal numbers of reference genes for accurate normalization. Different conditions are included and marked in square frames with different colors and the threshold used was 0.153336.

In the qRT-PCR, an RG may lead to biased results, while combining internal RGs can yield more precise and trustworthy gene expression data [1]. geNorm uses the relationship between the V and the cutoff value of 0.153336 to determine whether additional RGs need to be added to the RG set [17]. Ideally, the V_n_/V_n+1_ value should be less than 0.153336, i.e., the introduction of a new gene will not significantly affect the normalization [42]. Otherwise, n + 1 RGs need to be introduced. As shown in Figure 4, V_2_/V_3_ values were below 0.153336 for different organs, and different periods as well as all samples combined, demonstrating that adding a third internal RG had no discernible impact on the calibration of the qPCR data. Therefore, the optimal RG set was *eIF-2* + *His3.3* for both total samples and different periods, while the most suitable set of internal RGs was *TUA6* + *PP2A-3* in an organ subset.

#### 3.3.2. NormFinder Analysis

**Figure 5:**
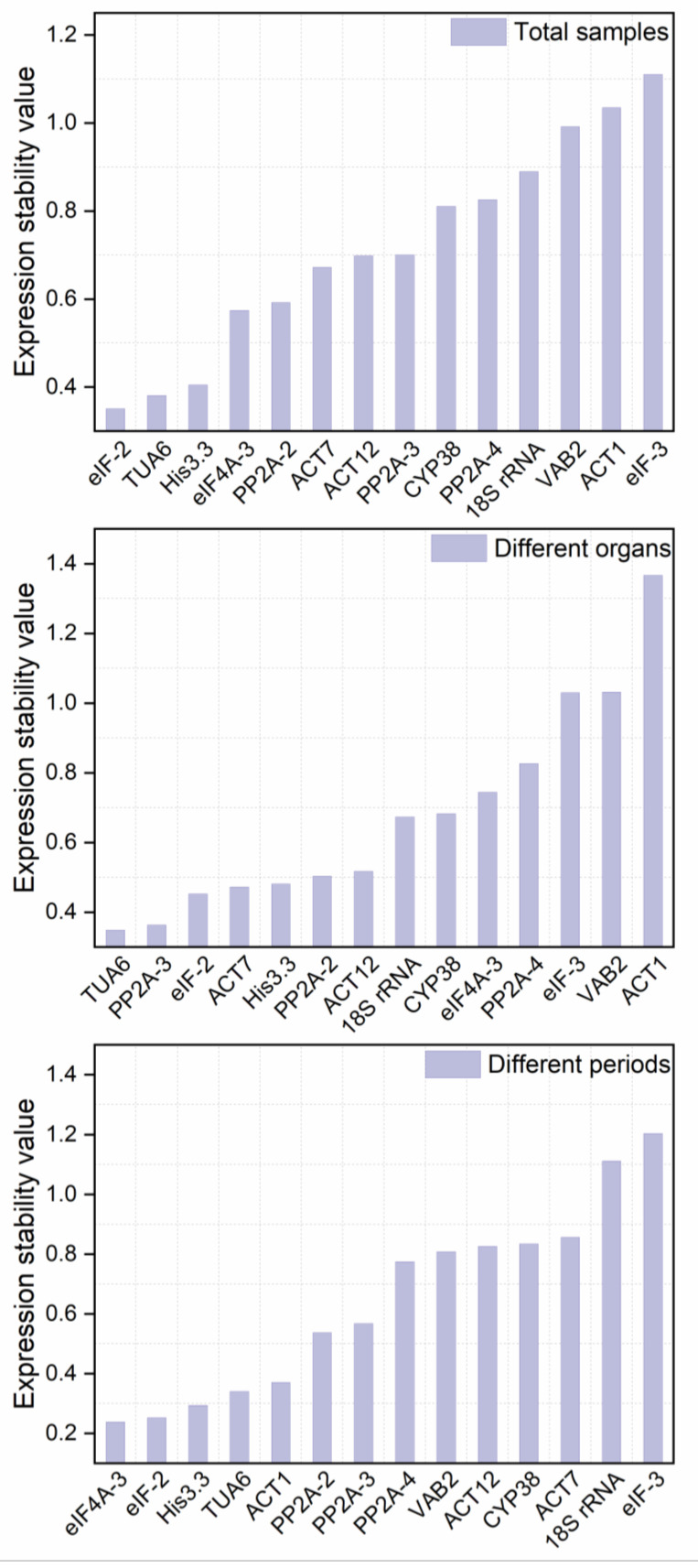
Expression stability of 14 reference genes in *P. vulgaris* analyzed by NormFinder.

The average pairwise variance of a gene in comparison to other RGs was calculated by the NormFinder to directly assess the stability of RGs [22]. Similar to geNorm, the NormFinder analysis also uses the 2^−ΔCt^ (ΔCT = each corresponding Ct value—the lowest Ct value for that gene) method [9]. In general, the lower the stability value (SV), the higher the expression stability of the corresponding gene. It can be seen from Figure 5 that the highest-ranked gene was not completely consistent under different experimental conditions. *eIF-2* and *eIF4A-3* were the most stable genes in all samples and different periods, respectively. Among the different organs, *TUA6* had the lowest stability value of 0.349. The results indicated that *TUA6* had the best stability among the 14 internal RGs in organs’ subsets, while *ACT1* (1.367) showed the highest variability. This agrees with the conclusions of the geNorm analysis. Interestingly, the worst-performing gene across all samples and different periods was *eIF-3*.

#### 3.3.3. BestKeeper Analysis

The BestKeeper software assesses the stability of internal RGs by directly calculating their coefficient of variation (CV), standard deviation (SD), and correlation coefficient (r) on the basis of each gene’s raw Ct value. It is worth noting that the most stable RG must have the highest r value, the lowest SD, and CV values [23]. It indicates that the expression of the RG is unstable and cannot be used for normalization when SD > 1 [23]. As shown in Table 2, the number of genes satisfying SD < 1 varied under different experimental conditions. Only *His3.3*, *eIF-2*, and *eIF4A-3* showed SD < 1 in organs’ subsets, which complied with the demands of internal RGs. Unfortunately, this was inconsistent with the findings of the geNorm and NormFinder analyses. *His3.3* (1.50 ± 0.33) and *eIF-2* (1.60 ± 0.34) were the highest-ranked among all samples and different periods, respectively. Interestingly, the BestKeeper analysis showed that the expression of *18S rRNA* was the least stable under different conditions and could not be used for normalization.

#### 3.3.4. Delta Ct Analysis

A Delta Ct analysis was used to calculate the mean standard deviation (SD) of each gene to determine the stability of the internal RGs. Higher genetic stability is associated with smaller SD [41]. With average SD values of just 0.374, *eIF-2* was the gene with the highest level of stability in organs’ subsets. The most stable RG was *His3.3* in both the total samples and the different periods, while the least stable gene was *VAB2*.

#### 3.3.5. RefFinder Analysis

An online analysis tool called RefFinder determined the geometric mean of each RG depending on the results of four statistical methods, Delta Ct, geNorm, NormFinder, and BestKeeper, to comprehensively rank the 14 candidate internal RGs. The smaller the geometric mean, the more consistent the expression of genes. The research found that *eIF-2* had the lowest indices in total data and different periods and was the most stable RG (Table 3), while *VAB2* and *18S rRNA* were the least stable genes under these two conditions, respectively. In contrast, *TUA6* performed the best, and *ACT1* the worst, in different organs. The differences in results between different software are understandable and acceptable due to the different assessment methods. Overall, in descending order, the stability of these 14 candidate RGs was: *eIF-2*, *His3.3*, *PP2A-2*, *TUA6*, *eIF4A-3*, *ACT7*, *ACT12*, *PP2A-3*, *PP2A-4*, *CYP38*, *eIF-3*, *18S rRNA*, *ACT1*, and *VAB2*.

### 3.4. RGs’ Validation

**Figure 6:**
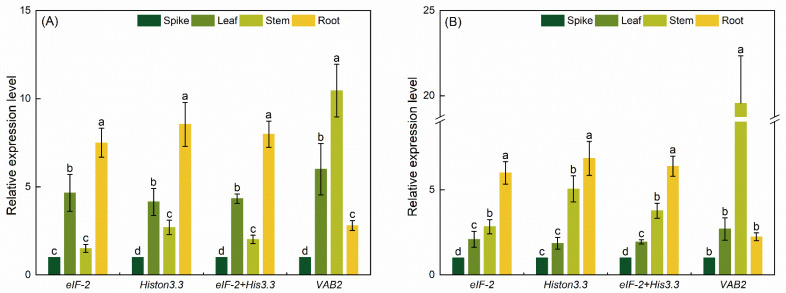
Validation of the identified reference genes. Relative expression of (**A**) *PvTAT* and (**B**) *Pv4CL2* in different organs of *P. vulgaris* that normalized by the most stable reference genes *eIF-2*, *His3.3*, *eIF-2 + His3.3*, and the least stable reference gene *VAB2*. The different letters signified significant differences at *p* < 0.05 by LSD test.

The previous study reported that the specific pattern of expression of *PvTAT* and *Pv4CL2*, genes involved in the control of rosmarinic acid production in *P. vulgaris*, was significantly distinct in different organs [45,46]. To confirm the accuracy of the identified internal RGs, the expression profiles of *PvTAT* and *Pv4CL2* in different organs of *P. vulgaris* were standardized by the two most reliable candidate RGs (*eIF-2* and *His3.3* alone or in combination) and the most unstable internal RGs (*VAB2*) from the above analysis. As shown in Figure 6, the expression levels of *PvTAT* and *Pv4CL2* normalized with *eIF-2*, *His3.3*, and the combination of *eIF-2*+*His3.3* showed perfect consistency in different organs. *PvTAT* was expressed at the highest level in roots (*p* < 0.05), then came the leaves, stems, and spikes. *Pv4CL2* had the highest significant expression in the roots (*p* < 0.05) and decreased from stems to leaves to spikes. In contrast, the expression level of *PvTAT* and *Pv4CL2* normalized with the worst internal reference gene *VAB2* and showed significant fluctuations. The relative expression level of *PvTAT* and *Pv4CL2* in the donor stems was significantly overestimated (*p* < 0.05), while the relative expression level in roots was obviously underestimated with incompatible expression patterns. In summary, the combination of *eIF-2* and *His3.3* should be the optimum RG set for the normalization of the qRT-PCR in diverse organs.

## 4. Discussion

In recent years, studying specific gene expression and regulatory mechanisms in various species has become an emerging hotspot [47]. The qPCR is a broadly accepted technique for gene expression analysis in molecular biology [48]. Due to the advantages of high sensitivity, high throughput, good reproducibility, and high specificity, the RT-qPCR technique has now become an important tool for gene expression studies in many laboratories [49]. Nevertheless, the accuracy of quantitative experimental results may be significantly impacted by variables somewhat like primer specificity, sample variation, and RNA quality [5]. During a gene expression analysis, internal RGs are often used to normalize the data to minimize or rectify errors generated during the quantification of target RGs. Consequently, to obtain accurate quantitative results, the selecting of appropriate internal RGs in a variety of experimental circumstances for a specific species is critical [7]. Internal RGs are genes that can be expressed as relatively stable in an organism under a particular condition. Commonly used RGs such as *ACT*, *UBQ*, *18S rRNA*, and *TUA* are usually genes that contribute to the preservation of cell structure or basic biochemical metabolic activities of the organism, and the expression stability of these genes is less affected by environmental factors. The ideal housekeeping genes must maintain relatively constant expression levels under different physiological conditions. Unfortunately, gene expression stability is influenced by many factors and varies in various organs or tissues, or at various growth and development phases. In other words, screening for stably expressed RGs under specific experimental conditions is crucial for analyzing qPCR results. *P. vulgaris* is a traditional Chinese medicine that was discovered and used as a medicinal herb thousands of years ago and has a high medicinal value. The medicinal part of *P. vulgaris* is mainly the dried and mature flower spike, which mainly contains rosmarinic acid, caffeic acid, ursolic acid, and other effective substances [29]. Nowadays, the research hotspots of *P. vulgaris* mainly focus on pharmacology and clinical application, and it is found to have therapeutic effects on various diseases including cancer [50,51]. Regrettably, the limited production of the active medicinal ingredients of *P. vulgaris* cannot meet the increasing market demand. Therefore, it is significant to exploit the genetic resources related to the biosynthesis of secondary metabolites and elucidate the molecular mechanism of Spica Prunellae development. Unfortunately, because of the absence of genetic information, the study on the molecular mechanism of *P. vulgaris* has been progressing slowly. Additionally, research on suitable RGs of *P. vulgaris* has also not been reported comprehensively.

In this research, 14 frequently employed candidate RGs (*18S rRNA*, *ACT1*, *ACT7*, *ACT12*, *eIF-3*, *eIF4A-3*, *eIF-2*, *His3.3*, *TUA6*, *CYP38*, *PP2A-2 PP2A-3*, *PP2A-4*, and *VAB2*) were screened based on *P. vulgaris* transcriptome data (GenBank accession: SRR7873856). Considering the differences in gene expression levels of the internal RGs in different organs and different developmental stages of the fruiting spike, it is necessary to combine more statistical tools to further analyze the expression stability of these candidate RGs under various circumstances. The different screening principles and focus of bioinformatics programs may lead to differences in the analysis results [52]. To get around the drawbacks of utilizing just a single analysis program, this study combined four commonly used Excel-based statistical algorithms, namely Delta Ct, geNorm, BestKeeper, and NormFinder, to assess the stability of internal RGs to improve the reliability of experimental data. Finally, the online software RefFinder was then used to rank each candidate internal RG in terms of the comprehensive index. It has been previously reported that in some cases, BestKeeper has a larger difference in results compared to other algorithms. For example, among all the samples of *D. styracifolium*, *SF3B* ranked first in the BestKeeper algorithm, while ranking low in other software [18]. In *I. indigotica*, BestKeeper ranked *ACT* as a medium, while geNorm and NormFinder methods ranked it as the most stable gene [20]. The same phenomenon also occurred in our study. *TUA6* ranked 11th in the BestKeeper analysis results of all samples, while it ranked 6th in Delta Ct, 2nd in NormFinder, 3rd in geNorm, and 4th in RefFinder. The geNorm analysis showed that among 14 genes in different organs of *P. vulgaris*, *TUA6* was the most suitable as RGs. This was consistent with NormFinder, but not the results of Delta Ct and BestKeeper. *eIF-2* and *His3.3* had lower SD and CV values and showed better stability in Delta Ct and BestKeeper analyses, respectively. Variations in the rankings of these algorithms among other species have also been reported [1,6,9,14,19,20], which may be because of the various underlying principles of the algorithms and the different screening focus used by the four analytical software, resulting in inconsistent results obtained even under the same conditions.

Some prior research has indicated that using a combination of RGs is preferable to using a single RG. RefFinder, an online tool integrating four algorithms, was used to comprehensively evaluate the candidate RGs. In addition, considering that the use of a single RG for normalization could produce inaccurate quantitative results, geNorm was used to calculate the V value to determine the optimal RG combination. It is noteworthy that the ideal V value must be less than the cutoff value of 0.153336 [17]. As shown in Figure 3, the pairwise variation values V_2_/V_3_ in different organs, different developmental periods, and all samples were smaller than the cutoff value. The results indicated that under these conditions, gene expression normalization can be accomplished without using the third internal RG. It was shown that the optimal combination of RGs in different organs was *TUA6* and *PP2A-2* among the 14 candidate RGs, while the optimal combination in different developmental periods and all samples was *eIF-2* and *His3.3*. All the results indicated the need to select the appropriate RGs according to the specific experimental conditions. Notably, the stability of *eIF-2* and *His3.3* genes was significantly better than that of other RGs under different conditions, while *ACT1* and *VAB2* were almost always ranked at the bottom of the stability list. So far, many prior studies have selected *eIF-2* and *His3.3* as internal RGs for standardization, such as *Nitraria tangutorum* [9] and *Ipomoea batatas* L. [53], etc.

As RGs, candidate genes with consistent expression levels, including *UBQ*, *ACT*, *18S rRNA*, and *GAPDH*, are commonly utilized in many studies [1,19,20]. However, the expression levels of these RGs vary among species and instances. RGs should be selected based on specific experiments. Even if a gene was shown to be expressed in a stable manner in numerous closely related species, it is crucial to confirm and validate the stability of the RG before applying it to gene expression normalization.

*PvTAT* is a key enzyme gene in the tyrosine branch of the rosmarinic acid metabolic pathway [45], which is intimately associated with the regulation of rosmarinic acid biosynthesis in *P. vulgaris*. *Pv4CL2* is one of the key enzymes involved in the phenylpropanoid pathway and also in the accumulation of rosmarinic acid [46]. The highest transcript abundances of *PvTAT* were found in roots and leaves, while the relatively lower transcript level was found in the stems. Furthermore, *PvTAT* showed the lowest expression levels in spikes [45]. The transcription of *Pv4CL2* was highest in roots and lower in stems, leaves, and spikes [46]. To confirm the accuracy of the experimental results even further, the expression patterns of the target genes *PvTAT* and *Pv4CL2* in different organs were detected and identified utilizing the two most stable genes, *eIF-2* and *His3.3*, and an unstable gene, *VAB2*, as RGs. The results showed that the expression patterns were almost identical, when *eIF-2*, *His3.3*, and *eIF-2* + *His3.3* were used to calibrate the expression of the target genes. However, when *VAB2* was used for normalization, its normalized expression patterns were incompatible with *eIF-2* + *His3.3* (Figure 6). These data again demonstrate that a reliable RG is a prerequisite for the correct normalization of target gene expression levels. Using inappropriate internal RGs can lead to the misestimation of target gene expression levels, resulting in the misinterpretation of experimental results. In conclusion, it is evident from this study that *eIF-2* + *His3.3* had the best stability in different developmental periods of spikes and total data, and they also had good stability in different organs, which has been verified. Therefore, these two RGs can be used as the optimal RG combinations of *P. vulgaris* for subsequent experimental studies.

## 5. Conclusions

The goal of this work was to identify the most suitable RGs for the qRT-PCR of mRNAs in different organs (root, stem, leaf, and spike) and Spica Prunellae developmental stages (bolting stage, early flowering stage, full flowering stage, and mature stage). Five popular algorithms (Delta Ct, geNorm, NormFinder, BestKeeper, and RefFinfer) were used to assess the expression stability of 14 typical candidate RGs in *P. vulgaris*. The results showed that the optimal RG and RG sets were *eIF-2* and *eIF-2* + *His3.3* in both different developmental stages and all samples, while *TUA6* and *TUA6* + *PP2A-2* were the most suitable RG and RG combinations in different organs. Therefore, in an RT-qPCR examination of this plant, they can be employed as RGs to normalize data depending on your demands. Furthermore, *VAB2*, *ACT1*, and *18S rRNA* showed low expression stability under different experimental conditions. The results of this study can help to accurately quantify the expression levels of target genes in *P. vulgaris* and provide guidance for obtaining reliable qPCR results subsequently, and also establish the groundwork for future research on the developmental mechanism of *P. vulgaris* flower spikes in the future.

## Figures and Tables

**Table 1:** Description of the fourteen candidate reference genes.

Gene Abbreviation	Gene ID	Gene Name	Arabidopsis Homolog Locus	Primer Sequences (5′–3′) (Forward/Reverse)	Tm (°C)	Length (bp)	Efficiency	R^2^
*ACT7*	SRR7873856.1.862141	Actin	AT5G09810	GTTACGAGCTTCCCGATGGA	59.54	193	105.56%	0.9989
				GATCCACCACTGAGCACGAT	59.82			
*ACT12*	SRR7873856.1.883268	Actin	AT3G46520	ACGGGTATCGTGCTTGACTC	59.83	182	108.36%	0.9957
				GAACAATTTCCGCTCGGCAG	60.18			
*ACT1*	SRR7873856.1.2050798	Actin	AT2G37620	GTCGGGACTGTGTGACTGAC	60.23	193	107.67%	0.9983
				CCCGGAAGAGCACCTAACTC	59.82			
*18S rRNA*	SRR7873856.1.1835427	18S ribosomal RNA	AT3G41768	GACGGAGGTAGGGTTCGATT	58.89	197	108.49%	0.9933
				CACCAGACTTGCCTCCAATG	58.83			
*eIF-3*	SRR7873856.1.968612	Translation initiation factor	AT4G20980	GGCTCTTGAGTCGCTCCAAT	60.11	195	107.04%	0.9993
				GCGAATCGTCGGTGTTCAAG	59.91			
*eIF4A-III*	SRR7873856.1.1836036	Translation initiation factor	AT3G19760	CCACCTTTTGCCTCCAACAC	59.61	191	106.06%	0.9971
				GGTACCGGGAAAACCTCCAT	59.38			
*eIF-2*	SRR7873856.1.18172	Translation initiation factor	AT1G76720	TTTTGGGAGAGCGGACACAA	59.82	196	102.96%	0.9927
				AGCTGCCTTGGAGACTGAAA	59.23			
*His3.3*	SRR7873856.1.2135040	Histone	AT4G40030	CACAAGGTAGGCCTCTGCTG	60.39	193	105.94%	0.9945
				AAGAAGCCCACAGATACCGC	60.11			
*TUA6*	SRR7873856.1.1877380	α-tubulin	AT4G14960	TCCACCCACTCCCTTCTTGA	60.10	193	105.31%	0.9960
				TTCATCCACGTTCAGGCTCC	60.04			
*CYP38*	SRR7873856.1.1584051	Cyclophilin	AT3G01480	CGCTCGAGAGGGTCGATAAC	60.04	188	102.00%	0.9921
				GCCTGCTACCACTTGACTGA	59.68			
*PP2A-2*	SRR7873856.1.2107443	Protein phosphatase 2Asubunit	AT1G10430	TTTAGATCAGAGGTGCGCGG	60.18	185	106.41%	0.9945
				AAATTGCTCTCGCGCCTGAT	60.75			
*PP2A-3*	SRR7873856.1.2002889	Protein phosphatase 2A subunit	AT2G42500	CCAGCACCTCGAGGGAGATA	60.47	182	103.48%	0.9971
				TTCCGACTGCACTGGTTGAA	59.82			
*PP2A-4*	SRR7873856.1.256172	Protein phosphatase 2A subunit	AT3G58500	GTGGCTTTGAAAGTGCGCTA	59.41	184	100.20%	0.9936
				TGATTCAACCAAGGCGGTCA	59.89			
*VAB2*	SRR7873856.1.1180932	Homeodomain transcription factor	AT3G05020	CTCGGAATTGTCGTCAGGCT	60.11	197	98.37%	0.9943
				ATCGTTGGCCGTTCAGGAAA	60.25			

**Table 2:** Stability analysis of 14 candidate reference genes based on BestKeeper software.

Rank	Total Samples				Different Organs				Different Periods			
	Gene	CV ± SD	r	*p*-Value	Gene	CV ± SD	r	*p*-Value	Gene	CV ± SD	r	*p*-Value
1	*His3.3*	1.50 ± 0.33	0.644	0.005	*His3.3*	1.42 ± 0.32	0.672	0.033	*eIF-2*	1.60 ± 0.34	0.910	0.001
2	*eIF-2*	1.76 ± 0.38	0.926	0.001	*eIF-2*	1.79 ± 0.39	0.937	0.001	*His3.3*	1.93 ± 0.43	0.799	0.010
3	*eIF4A-3*	2.85 ± 0.66	0.663	0.004	*eIF4A-3*	3.05 ± 0.71	0.573	0.083	*PP2A-2*	1.95 ± 0.45	0.208	0.593
4	*PP2A-2*	3.30 ± 0.77	0.489	0.046	*CYP38*	4.34 ± 1.18	0.861	0.001	*eIF4A-3*	2.23 ± 0.51	0.836	0.005
5	*ACT1*	3.32 ± 1.15	0.321	0.210	*eIF-3*	4.41 ± 1.08	0.306	0.389	*ACT7*	2.56 ± 0.59	0.513	0.810
6	*eIF-3*	3.77 ± 0.91	0.028	0.914	*ACT1*	4.46 ± 1.54	0.208	0.565	*ACT1*	2.88 ± 1.01	0.950	0.001
7	*ACT7*	3.97 ± 0.91	0.527	0.030	*PP2A-2*	4.52 ± 1.07	0.810	0.004	*ACT12*	3.24 ± 0.71	0.108	0.780
8	*CYP38*	4.66 ± 1.28	0.699	0.002	*PP2A-3*	4.62 ± 1.06	0.934	0.001	*eIF-3*	3.48 ± 0.83	0.534	0.055
9	*ACT12*	4.71 ± 1.02	0.454	0.068	*ACT7*	4.71 ± 1.07	0.864	0.001	*PP2A-3*	4.52 ± 1.12	0.924	0.001
10	*PP2A-3*	4.93 ± 1.17	0.907	0.001	*TUA6*	4.99 ± 1.02	0.808	0.005	*TUA6*	4.59 ± 0.94	0.889	0.001
11	*TUA6*	4.94 ± 1.01	0.837	0.001	*ACT12*	5.79 ± 1.25	0.745	0.013	*CYP38*	5.08 ± 1.44	0.709	0.032
12	*PP2A-4*	6.51 ± 1.49	0.998	0.001	*PP2A-4*	7.43 ± 1.67	0.992	0.001	*PP2A-4*	6.81 ± 1.62	0.995	0.001
13	*VAB2*	7.43 ± 1.68	0.995	0.001	*VAB2*	8.99 ± 1.99	0.995	0.001	*VAB2*	7.01 ± 1.67	0.999	0.001
14	*18S rRNA*	19.52 ± 1.54	0.895	0.001	*18S rRNA*	15.67 ± 1.27	0.944	0.001	*18S rRNA*	24.88 ± 2.04	0.931	0.001

**Table 3:** Comprehensive evaluation of expression stability of fourteen candidate reference genes in *P. vulgaris* using the RefFinder algorithm.

Group	Rank	Delta Ct	BestKeeper	NormFinder	geNorm	RefFinder	
		Gene	Gene	Gene	Gene	Gene	SV
Total samples	1	*His3.3*	*His3.3*	*eIF-2*	*eIF-2*	*eIF-2*	1.190
	2	*eIF-2*	*eIF-2*	*TUA6*	*His3.3*	*His3.3*	1.570
	3	*eIF4A-3*	*eIF4A-3*	*His3.3*	*TUA6*	*PP2A-2*	3.940
	4	*PP2A-2*	*PP2A-2*	*eIF4A-3*	*PP2A-2*	*TUA6*	3.980
	5	*ACT7*	*ACT1*	*PP2A-2*	*eIF4A-3*	*eIF4A-3*	4.530
	6	*TUA6*	*eIF-3*	*ACT7*	*ACT7*	*ACT7*	5.180
	7	*ACT12*	*ACT7*	*ACT12*	*ACT12*	*ACT12*	6.650
	8	*eIF-3*	*CYP38*	*PP2A-3*	*PP2A-3*	*PP2A-3*	8.460
	9	*ACT1*	*ACT12*	*CYP38*	*PP2A-4*	*PP2A-4*	9.930
	10	*PP2A-3*	*PP2A-3*	*PP2A-4*	*CYP38*	*CYP38*	9.970
	11	*CYP38*	*TUA6*	*18S rRNA*	*18S rRNA*	*eIF-3*	10.920
	12	*18S rRNA*	*PP2A-4*	*VAB2*	*VAB2*	*18S rRNA*	11.470
	13	*PP2A-4*	*VAB2*	*ACT1*	*eIF-3*	*ACT1*	12.310
	14	*VAB2*	*18S rRNA*	*eIF-3*	*ACT1*	*VAB2*	12.470
Different organs	1	*eIF-2*	*His3.3*	*TUA6*	*TUA6*	*TUA6*	1.410
	2	*His3.3*	*eIF-2*	*PP2A-3*	*PP2A-3*	*PP2A-2*	3.220
	3	*PP2A-2*	*eIF4A-3*	*eIF-2*	*PP2A-2*	*eIF-2*	3.310
	4	*eIF4A-3*	*CYP38*	*ACT7*	*eIF-2*	*PP2A-3*	3.440
	5	*ACT7*	*eIF-3*	*His3.3*	*ACT7*	*His3.3*	3.810
	6	*ACT12*	*ACT1*	*PP2A-2*	*ACT12*	*ACT7*	4.860
	7	*eIF-3*	*PP2A-2*	*ACT12*	*His3.3*	*ACT12*	5.960
	8	*TUA6*	*PP2A-3*	*18S rRNA*	*18S rRNA*	*eIF4A-3*	7.000
	9	*ACT1*	*ACT7*	*CYP38*	*CYP38*	*18S rRNA*	8.920
	10	*PP2A-3*	*TUA6*	*eIF4A-3*	*eIF4A-3*	*CYP38*	9.240
	11	*CYP38*	*ACT12*	*PP2A-4*	*PP2A-4*	*eIF-3*	10.610
	12	*PP2A-4*	*PP2A-4*	*eIF-3*	*eIF-3*	*PP2A-4*	11.720
	13	*VAB2*	*VAB2*	*VAB2*	*VAB2*	*VAB2*	13.240
	14	*18S rRNA*	*18S rRNA*	*ACT1*	*ACT1*	*ACT1*	13.470
Different periods	1	*His3.3*	*eIF-2*	*eIF4A-3*	*eIF-2*	*eIF-2*	1.190
	2	*eIF-2*	*His3.3*	*eIF-2*	*His3.3*	*His3.3*	1.860
	3	*eIF4A-3*	*PP2A-2*	*His3.3*	*eIF4A-3*	*eIF4A-3*	2.450
	4	*TUA6*	*eIF4A-3*	*TUA6*	*TUA6*	*PP2A-2*	4.560
	5	*PP2A-2*	*ACT7*	*ACT1*	*ACT1*	*TUA6*	5.470
	6	*ACT7*	*ACT1*	*PP2A-2*	*PP2A-2*	*ACT1*	6.510
	7	*eIF-3*	*ACT12*	*PP2A-3*	*PP2A-3*	*ACT7*	7.580
	8	*ACT12*	*eIF-3*	*PP2A-4*	*PP2A-4*	*ACT12*	7.750
	9	*PP2A-3*	*PP2A-3*	*VAB2*	*VAB2*	*PP2A-3*	8.150
	10	*CYP38*	*TUA6*	*ACT12*	*ACT12*	*PP2A-4*	9.360
	11	*18S rRNA*	*CYP38*	*CYP38*	*ACT7*	*VAB2*	10.370
	12	*ACT1*	*PP2A-4*	*ACT7*	*CYP38*	*CYP38*	11.490
	13	*PP2A-4*	*VAB2*	*18S rRNA*	*18S rRNA*	*eIF-3*	11.770
	14	*VAB2*	*18S rRNA*	*eIF-3*	*eIF-3*	*18S rRNA*	13.240

## Data Availability

The data presented in this study are available in Supplementary Materials.

## Supplementary Materials

The following supporting information can be downloaded at: https://www.mdpi.com/article/10.3390/genes13111947/s1, Figure S1: qPCR amplification specificity of 14 candidate reference genes. Amplification fragments were separated by 1.5% agarose gel electrophoresis; Figure S2: Melt curves for the 14 candidate reference genes; Figure S3: Standard curves of the 14 candidate reference genes; Table S1: Cq values of 14 candidate reference genes in all *P. vulgaris* samples.
