# Supplementary material for: Systematic Identification and Validation of Suitable Reference Genes for the Normalization of Gene Expression in Prunella vulgaris under Different Organs and Spike Development Stages
Source: Genes (Basel). 2022 Oct 25;13(11):1947. doi: 10.3390/genes13111947 (PMC9689956; doi:10.3390/genes13111947)
Supplement: Supplementary file 1 [file genes-13-01947-s001.zip › Supplementary Figure.pdf]

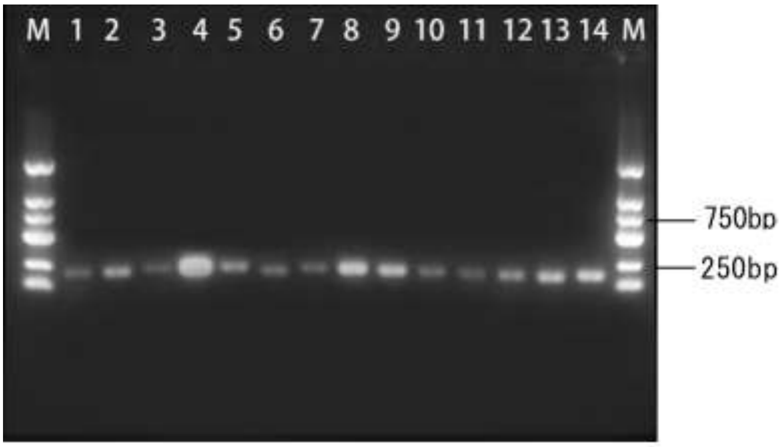

Figure S1. qPCR amplification specificity of 14 candidate reference genes. Amplification fragments were separated by 1.5% agarose gel electrophoresis.
1: *ACT7;* 2: *ACT12;* 3: *ACT1*; 4: *18S rRNA*; 5: *eIF-3*; 6*: eIF4A-3*; 7: *eIF-2*; 8: *His3.3*; 9: *TUA6*; 10: *CYP38*; 11: *PP2A-2*; 12: *PP2A-3*; 13: *PP2A-4*; 14: *VAB2*; M: Marker 2K.

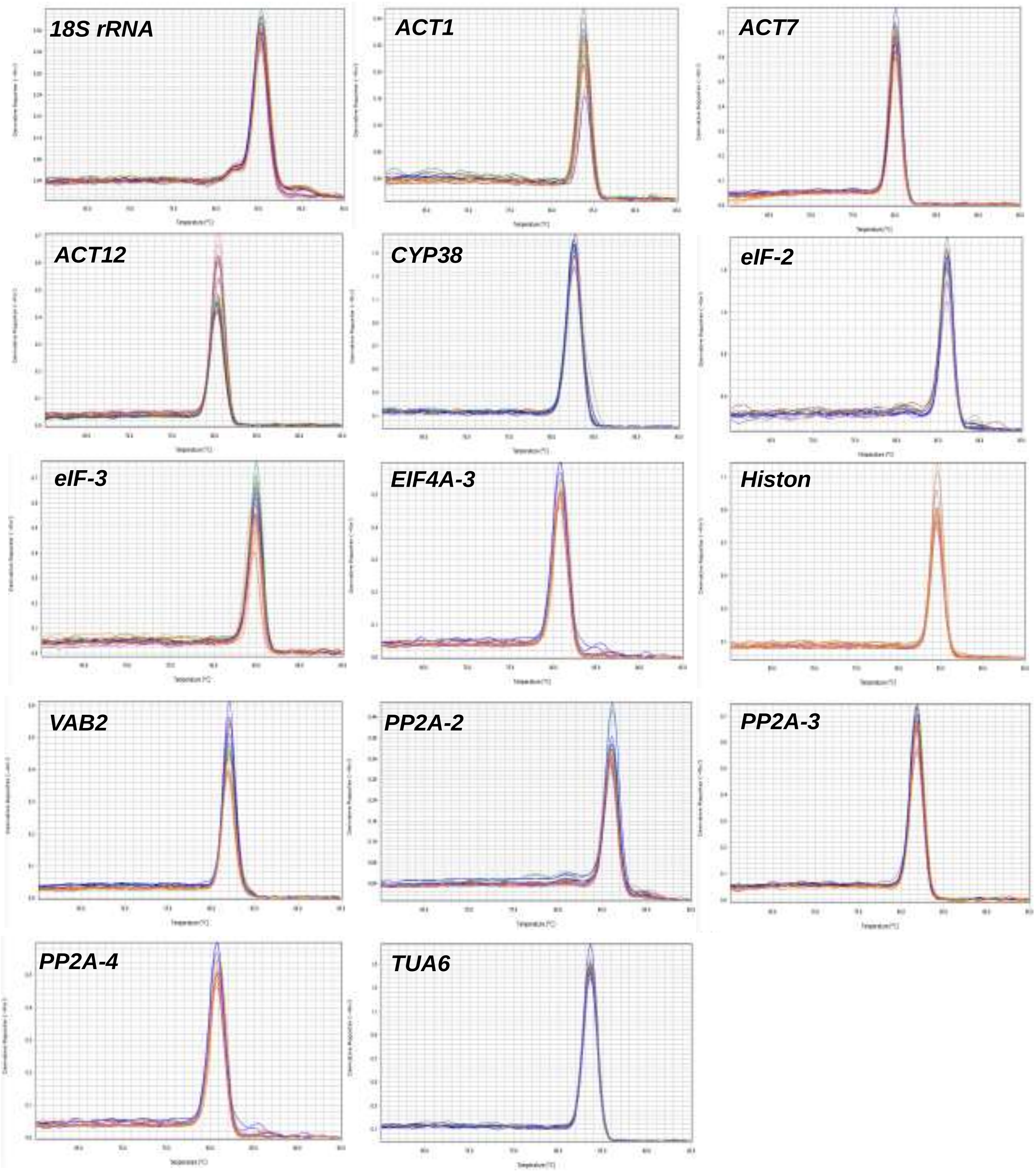

Fig. S2 Melt curves for the 14 candidate reference genes

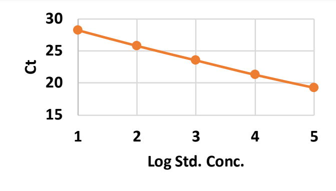

ACT7	
R^2	0.9989
Slope	-2.2335
Y-Intercept	30.298
PCR Efficiency	105.56%

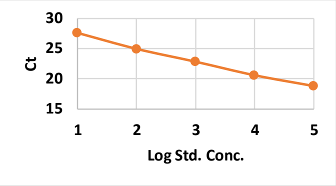

ACT12	
R^2	0.9957
Slope	-2.1924
Y-Intercept	29.521
PCR Efficiency	108.36%

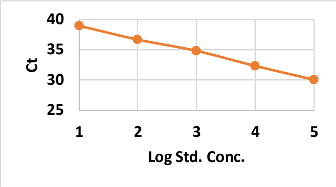

ACT1	
R^2	0.9983
Slope	-2.2024
Y-Intercept	41.14
PCR Efficiency	107.67%

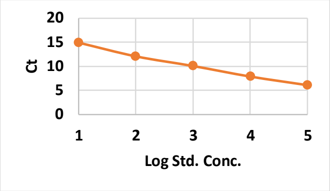

18S rRNA	
R^2	0.9933
Slope	-2.1905
Y-Intercept	16.763
PCR Efficiency	108.49%

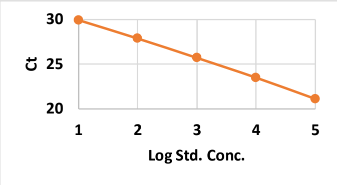

eIF-3	
R^2	0.9993
Slope	-2.2116
Y-Intercept	32.233
PCR Efficiency	107.04%

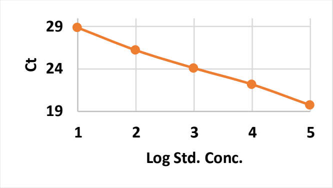

EIF4A-3	
R^2	0.9971
Slope	-2.226
Y-Intercept	30.88
PCR Efficiency	106.06%

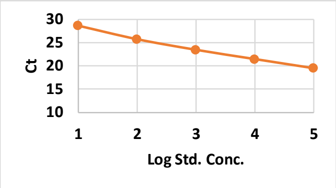

eIF-2	
R^2	0.9927
Slope	-2.2738
Y-Intercept	30.526
PCR Efficiency	102.96%

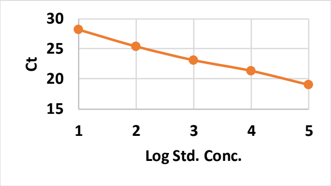

His3.3	
R^2	0.9945
Slope	-2.2278
Y-Intercept	30.096
PCR Efficiency	105.94%

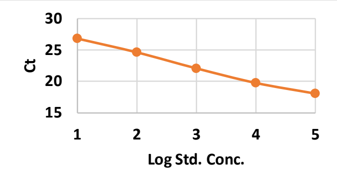

TUA6	
R^2	0.996
Slope	-2.2374
Y-Intercept	28.967
PCR Efficiency	105.31%

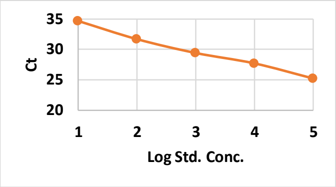

CYP38	
R^2	0.9921
Slope	-2.2891
Y-Intercept	36.618
PCR Efficiency	102.00%

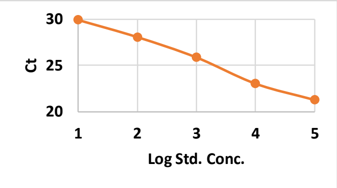

PP2A-2	
R^2	0.9945
Slope	-2.2209
Y-Intercept	32.281
PCR Efficiency	106.41%

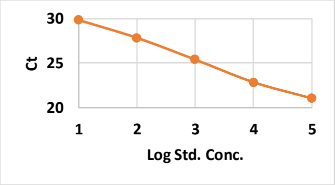

PP2A-3	
R^2	0.9971
Slope	-2.2656
Y-Intercept	32.166
PCR Efficiency	103.48%

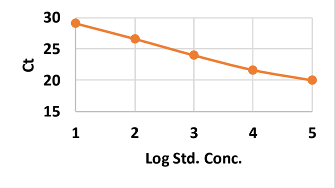

PP2A-4	
R^2	0.9936
Slope	-2.3186
Y-Intercept	31.175
PCR Efficiency	100.20%

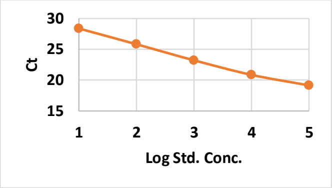

VAB2	
R^2	0.9943
Slope	-2.3496
Y-Intercept	30.511
PCR Efficiency	98.37%

Fig. S3 Standard curves of the 14 candidate reference
